# The Impact on Inpatient Stays, Crisis and Emergency Department Assessments In Patients With Emotionally Unstable Personality Disorder Who Complete an 18-Month Mentalization-Based Therapy Programme in a Tertiary Personality Disorder Service in Northern Ireland

**DOI:** 10.1192/bjo.2024.366

**Published:** 2024-08-01

**Authors:** Adam Flynn, Owen McNeill, Chris Walsh, Cedar Andress

**Affiliations:** 1 NIMDTA, Belfast, United Kingdom.; 2 Northern Health & Social Care Trust, Antrim, United Kingdom

## Abstract

**Aims:**

The Personality Disorder Service in the Northern Health & Social Care Trust was originally set up to deliver evidence-based treatment for people with the diagnosis of personality disorder. This group of people historically have been stigmatised, excluded and let down by services, despite their complex needs and frequent history of childhood trauma. The team developed a Mentalization Based Therapy (MBT) programme originally commencing in 2013.

To identify recent completers of the MBT 2 18 month programme and to assess whether there was any reduction or change in pattern to the number of days spent as inpatient both during and after having completed the programme, whether there was a reduction in the frequency of same day assessments with community mental health teams or unscheduled care and finally whether there was any reduction in terms of volume of crisis assessments and presentations to Emergency Department.

**Methods:**

Using validated Quality Improvement Methods, a Plan Do Study Act Cycle was commenced which involved identifying patients who had begun and finished the MBT programme and minimum of 12 months had passed since completion in order to follow-up.

We then broke down this data into 3 domains. By using EPEX, Paris and Electronic Care Record computer systems, it was possible to analyse days spent as inpatient, same day assessments and crisis assessments as well as Emergency Department attendance.

For these periods of time, they were split into pre-commencement of programme (18 months), during programme (18 months) and post-completion of programme (12 months) to see if there was any tangible decrease in these numbers.

19 service users were identified that had initially been referred to Personality Disorder Service between 2016 and 2018 and who subsequently began MBT2 programme between 2017 and 2019. Given the length of completion of the programme, this allowed us to gather a full set of data with regard to these patients up to completion of programme in 2021. Subsequent period of 12 months was then analysed post-completion of treatment taking us up to 2022.

**Results:**

The average time spent in inpatient admission days prior to starting therapy for 18 months (n = 19) was 21.74 days, this decreased to 6.53 during therapy and 3.68 post-therapy (12 month follow-up) = 5.52 adjusted for 18 months. This represents a reduction of 74.61%.

The average number of same day assessments and unscheduled care (n = 8) seeking prior to admission was 1.38. This decreased to 0.75 during therapy and 0.88 post-therapy adjusted to 1.32 for 18 months, which represents a small decline of 4.35%.

Finally, the average number of Crisis contacts and Emergency Department assessments were 2.63 in the 18 months before commencing therapy, 1.26 during therapy and 0.58 in the 12 months post-therapy, 0.87 adjusted for 18 months. This represents a reduction of 66.92%

**Conclusion:**

It is clear from analysis of the data that there has been a substantial decrease in time spent as admitted inpatient as well as number of contacts with Crisis Assessors and Emergency Departments in association with completion of the MBT 18 month programme.

This demonstrates that, by using an evidence-based and well-established programme, which carries a high time commitment for both service users and practitioners, it is possible to considerably reduce use of other, more acute services and keep patients with a diagnosis of EUPD out of hospital longer and on a sustained basis and also to reduce presentations to Emergency Departments which was often on the basis of self-harm and/or overdoses.

The dual result is that it can be validated objectively that service users are suffering less distress after having completed the programme, which will lead to better quality of life, whilst also reducing the burden on costly inpatient services with the end result being an important investment in mental health services in Northern Ireland and the prototype for the developing regional service.

